# Mesenchymal Stem Cell‐Derived Exosomes as a Double‐Edged Sword: Balancing Inflammation and Immunosuppression in Human Papillomavirus‐Infected Tissues

**DOI:** 10.1155/sci/2596453

**Published:** 2026-07-19

**Authors:** Fatemeh Poorhoseini Hanzaii, Masoud Soleimani, Mina SoufiZomorrod

**Affiliations:** ^1^ Applied Cell Sciences Division, Department of Hematology, Faculty of Medical Sciences, Tarbiat Modares University, Tehran, Iran, modares.ac.ir; ^2^ Department of Tissue Engineering and Applied Cell Sciences, School of Advanced Technologies in Medicine, Shahid Beheshti University of Medical Sciences, Tehran, Iran, sbmu.ac.ir

**Keywords:** exosomes, HPV, immunomodulation, inflammation, mesenchymal stem cells

## Abstract

Human papillomavirus (HPV) infection is the most common sexually transmitted viral infection, strongly associated with chronic inflammation and cervical cancer progression in women. Persistent HPV infection leads to an inflammatory microenvironment that promotes epithelial dysplasia and immune evasion. Exosomes derived from mesenchymal stem cells (MSC‐exosomes) have emerged as promising immunomodulatory and anti‐inflammatory agents. This review examines current evidence on the interaction between HPV‐induced inflammation and exosomal signaling, with a particular focus on the therapeutic potential of MSC‐exosomes. We discuss their roles in immune regulation, miRNA delivery, suppression of nuclear factor kappa‐light‐chain‐enhancer of activated B cells (NF‐κB) signaling, and epithelial regeneration. This anti‐inflammatory effect may also impair local immune surveillance, potentially enabling viral persistence and progression to malignancy. In this narrative review, We reviewed PubMed, Scopus, and Web of Science articles published up to 2024 on MSC‐exosome interactions with immune regulation in HPV‐related diseases. Evidence suggests that while the anti‐inflammatory effects of MSC‐exosomes may help control HPV‐associated inflammation, they can also impair local immune surveillance, potentially facilitating viral persistence and progression toward malignancy. This dual activity positions MSC‐exosomes as a double‐edged sword in the context of HPV pathogenesis. A deeper understanding of this paradox is essential for designing safer, context‐specific MSC‐based therapies that balance anti‐inflammatory benefits with effective antiviral immune responses. Although direct studies on MSC‐exosomes in HPV infections are limited, existing models suggest that they have the capacity to attenuate inflammation and restore cervical tissue homeostasis. This article also highlights knowledge gaps and future research directions necessary to develop MSC‐exosome‐based therapies for HPV‐related cervical diseases.

## 1. Introduction

### 1.1. HPV Biology and Pathogenesis

Human papillomavirus (HPV) is a nonenveloped, double‐stranded DNA virus with a distinct tropism for epithelial tissues, particularly those of the anogenital tract, oropharynx, and cutaneous surfaces. HPV is recognized as the most prevalent sexually transmitted infection (STI) globally [1, 2]. Although the majority of infections are transient and are spontaneously cleared by the host immune system within 12–24 months, persistent infection with high‐risk genotypes poses a significant risk for the development of high‐grade cervical intraepithelial neoplasia (CIN) 2/3 and eventual progression to invasive carcinoma, particularly in immunocompromised individuals [3]. Over 200 genotypes of HPV have been identified to date, broadly categorized into low‐risk types—such as HPV‐6 and HPV‐11, associated with benign lesions like genital warts—and high‐risk types—such as HPV‐16 and HPV‐18, which are strongly linked to premalignant conditions and various cancers, including cervical, anal, vulvar, penile, and oropharyngeal carcinomas [4]. HPV infection typically occurs through microabrasions in mucosal or cutaneous epithelia, allowing the virus to access and infect the basal keratinocytes [5]. Once inside the host cell, HPV exploits the natural differentiation process of epithelial cells to complete its life cycle and maintain persistence [6].

The pathogenesis of HPV‐related diseases is strongly influenced by the host immune response and the chronic inflammatory microenvironment induced by the virus [7]. Persistent infection with high‐risk genotypes, notably HPV‐16 and HPV‐18, fosters a local immunosuppressive milieu that facilitates viral persistence and the progression of epithelial dysplasia. HPV initiates infection by binding to epithelial cell surface heparan sulfate proteoglycans and other cell‐specific receptors, facilitating entry via both clathrin‐dependent and ‐independent endocytic pathways. Upon entry, the circular double‐stranded HPV genome is established as a stable episome in some cells of the basal epithelial layer [7]. HPV exhibits a strong tropism for epithelial tissues, particularly the stratified squamous epithelium. The virus specifically targets basal epithelial cells, which are located in the lowest layer of this epithelium and are responsible for cellular regeneration and repair. These cells are found in various anatomical sites, including the cervix (especially the transformation zone), vulva, vagina, anus, penis, and oropharyngeal region [1]. Once HPV enters the basal layer, it establishes infection by introducing its DNA into the host cell nucleus, often remaining latent or undetected by the immune system. The virus relies on the natural differentiation process of the epithelial cells to complete its life cycle, producing new viral particles as the infected cells move toward the surface [8].

### 1.2. HPV Infection and the Role of Epithelial Adult Tissue Stem Cells

**Figure 1:**
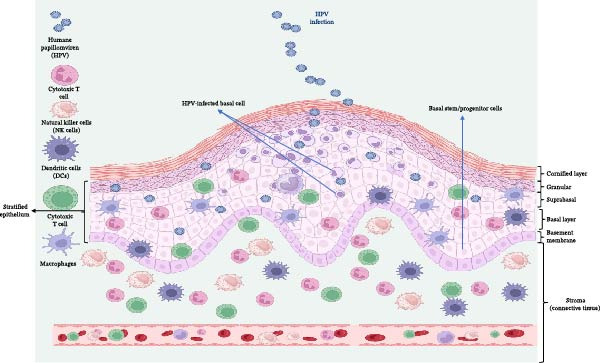
HPV invasion into epithelial cells and subsequent immune cell activation.

Although HPV infection of basal epithelial cells is generally considered a nonselective event occurring at sites of epithelial microabrasion, increasing evidence suggests that, by chance, the virus may infect adult epithelial stem or early progenitor cells residing within the basal layer [9, 10] (Figure 1). These long‐lived stem cells, characterized by their self‐renewal capacity and a central role in epithelial homeostasis, represent a potentially critical reservoir for persistent viral maintenance. Once established in epithelial stem cells, HPV genomes can be maintained at low copy numbers in an episomal form, promoting viral latency and immune evasion. During stem cell division, viral episomes are passively segregated into daughter cells, enabling long‐term persistence without continuous productive replication [11]. Because these stem cells give rise to both progenitor and differentiated epithelial cells, infection at this level may generate a mosaic of genetically and epigenetically altered cells across a broad epithelial region, creating a pre‐malignant “field” rather than a single focal lesion [12]. Importantly, conventional antiviral and immune‐mediated mechanisms preferentially eliminate actively replicating virus in differentiated epithelial cells, whereas latent HPV genomes residing within quiescent or slowly cycling stem cell populations largely evade immune surveillance [13]. Therefore, as the process of infection in stem and progenitor cells, leading to intraepithelial neoplasia (IEN), progresses, normal terminal differentiation is disrupted. Since expression of HPV requires differentiation, expression of early genes in basal cells and late genes such as L1 and L2 in fully differentiated suprabasal keratinocytes, such disruption will inevitably result in altered late gene expression, leading to a decrease in virus production and the accumulation of infected undifferentiated cells [14]. For persistent infection to be maintained within the tissue, these HPV‐infected stem cells proliferate beyond their normal niche, contributing to the pathogenesis of HPV‐associated lesions. This protected stem cell reservoir constitutes a fundamental barrier to durable viral clearance, promoting viral latency, reactivation, lesion recurrence, and the delayed onset characteristic of HPV‐associated carcinogenesis [12]. Consequently, therapies targeting only differentiated cells may provide transient benefits without eradicating the underlying stem cell–associated viral reservoir [13]. The disruption of epithelial differentiation not only impairs productive HPV infection but also allows the virus to persist in stem and progenitor cells. This protected reservoir constitutes a fundamental barrier to durable viral clearance, contributing to viral latency, reactivation, lesion recurrence, and the delayed onset characteristic of HPV‐associated carcinogenesis. Consequently, therapies targeting only differentiated cells may provide transient benefits without eradicating the underlying stem cell–associated viral reservoir [12].

### 1.3. Mechanisms of HPV‐Mediated Immune Escape

HPV has developed multiple sophisticated mechanisms to evade host immune responses, thereby promoting persistent infection and long‐term survival within epithelial tissues. Among the most critical immune evasion mechanisms is the targeted disruption of antigen presentation pathways, particularly through the downregulation of major histocompatibility complex class I (MHC‐I) molecules on the surface of infected cells. By impairing the presentation of endogenously derived viral peptides to CD8^+^ CTLs, HPV effectively reduces immune recognition and cytolytic clearance of infected keratinocytes [15]. This downregulation is primarily mediated by the HPV early proteins E5, E6, and E7, each contributing to the suppression of antigen presentation through distinct molecular mechanisms. HPV employs several oncoproteins to evade immune detection, particularly by impairing MHC‐I–mediated antigen presentation. The E5 protein hinders MHC‐I transport to the cell surface by retaining it in intracellular compartments. E7 downregulates key components of the antigen‐processing machinery, including transporter associated with antigen processing 1, 2 (TAP1/2) and low molecular weight protein 2, 7 (LMP2/7), and disrupts interferon‐stimulated gene (ISG) expression via the inactivation of pRb. Collectively, these mechanisms enable HPV to escape CTL surveillance by interfering with multiple steps of the antigen presentation pathway [16]. HPV effectively evades host immunity by targeting key components of the interferon (IFN) signaling pathways. HPV oncoproteins E6 and E7 evade host antiviral responses by disrupting IFN signaling. E6 inhibits IFN regulatory factor 3, 7 (IRF3/7) and degrades p53, impairing ISG transcription, while E7 blocks STAT1/2 activation, suppressing JAK‐STAT signaling. This reduces MHC expression and cytotoxic T cell activity, enabling viral persistence [17]. Persistent suppression of antigen presentation not only facilitates viral latency but also impairs the immunogenicity of transformed cells, thereby creating an immune‐privileged environment that supports malignant progression and poses a substantial barrier to immunotherapeutic intervention [18]. In addition, HPV interferes with cytokine signaling by modulating IFN pathways and inducing the expression of immunosuppressive cytokines such as IL‐10 and transforming growth factor‐beta (TGF‐β) [19]. Unlike many other viruses that enter the bloodstream and cause systemic viremia, HPV infection remains entirely confined to the epithelium. This spatial restriction has profound immunological consequences: by avoiding viremia, HPV effectively minimizes its exposure to systemic immune surveillance mechanisms, including circulating antigen‐presenting cells (APCs), dendritic cells, and CTLs [20]. Furthermore, HPV does not induce cytolysis or robust inflammation during its replication cycle, thereby limiting the release of damage‐associated molecular patterns (DAMPs) that would normally activate innate immune responses. The virus completes its life cycle in synchrony with the differentiation program of keratinocytes, from basal layer infection through to virion assembly and shedding in the upper layers of the epithelium—compartments that are largely devoid of immune cells [21]. This highly compartmentalized infection strategy enables HPV to persist undetected for extended periods, promoting chronicity and increasing the risk of malignant transformation, especially in high‐risk HPV types such as HPV‐16 and HPV‐18. Chronic inflammation sustains activation of nuclear factor kappa‐light‐chain‐enhancer of activated B cells (NF‐κB), STAT3, and cyclooxygenase‐2 (COX‐2), promoting viral persistence and upregulating matrix metalloproteinases (MMPs) and pro‐angiogenic factors like vascular endothelial growth factor (VEGF), which drive ECM degradation, neovascularization, and epithelial disorganization [22]. Concurrently, pro‐inflammatory cytokines (IL‐6, TGF‐β, and TNF‐α) activate epithelial–mesenchymal transition (EMT) transcription factors such as snail, twist, and zinc finger E‐box binding homeobox 1 (ZEB1), leading to loss of epithelial markers (E‐cadherin) and gain of mesenchymal traits (vimentin and N‐cadherin), enhancing motility, invasion, and apoptosis resistance [23]. Collectively, chronic inflammation remodels the tissue microenvironment to support HPV persistence and carcinogenesis [24]. Therefore, the host immune system’s inability to clear the virus, combined with persistent inflammatory signaling, plays a central role in HPV‐associated carcinogenesis. Inflammation plays a dual role in HPV infection [25]. Inflammation plays a complex and dual role in the context of HPV infection. On one hand, it represents a critical component of the innate and adaptive immune response, initiating the recruitment of immune cells and the production of antiviral cytokines aimed at eliminating the virus. Acute inflammation, when properly regulated, facilitates viral clearance and limits tissue damage. However, when inflammation becomes chronic or dysregulated—as frequently observed in persistent high‐risk HPV infections—it shifts from a protective response to a pathological one [26]. HPV infects epithelial cells and triggers immune cell activation (Figure 1).

### 1.4. Overview of Current HPV Treatments and Remaining Challenges

Over the years, various therapeutic strategies have been developed to manage HPV infections and associated inflammation, yet several challenges remain. The overview of the main treatment modalities and the reasons for their limited efficacy are shown later in this study.

#### 1.4.1. Antiviral Agents: Cidofovir and IFNs

Cidofovir and IFNs have been studied for treating HPV‐associated diseases due to their antiviral and immunomodulatory effects. Cidofovir inhibits viral DNA replication and downregulates E6/E7 oncogene expression, showing efficacy in reducing genital warts, especially in immunocompromised individuals. However, its clinical use is limited by nephrotoxicity, local side effects, and the inability to eliminate latent HPV. IFNs (notably IFN‐α and IFN‐β) enhance immune responses by activating natural killer (NK) cells and increasing MHC‐I expression, but clinical outcomes have been inconsistent, with systemic side effects and reduced efficacy in immunosuppressive environments. Overall, both agents face limitations in bioavailability, tolerability, and clearance of persistent infection [27–29].

#### 1.4.2. Immunomodulatory Therapies: Imiquimod and Related Agents

Imiquimod, a TLR7 agonist, enhances innate and adaptive immune responses by inducing cytokine production and promoting cytotoxic T cell activity against HPV‐infected cells. It is clinically approved for external genital and perianal warts, with variable efficacy (35%–75%) depending on lesion characteristics and host immunity. However, prolonged treatment, local adverse effects, and limited activity against high‐risk HPV types and latent infections reduce its overall effectiveness. Other investigational agents, such as resiquimod (TLR7/8 agonist) and poly‐ICLC (a synthetic dsRNA), have shown potential in enhancing antiviral immunity [25]. These agents function by activating innate immune pathways, promoting type I IFN production, and stimulating dendritic cell maturation and cytotoxic T lymphocyte responses. Despite encouraging results in preclinical models and early‐phase clinical trials, both compounds have yet to demonstrate consistent therapeutic efficacy in large‐scale, randomized human studies. Limitations such as dosing challenges, local and systemic tolerability, and variable immunogenic responses across patient populations continue to hinder their clinical translation. Further research is warranted to optimize delivery strategies and identify patient subsets most likely to benefit from these novel immunomodulators [30].

#### 1.4.3. Surgical and Ablative Procedures: Efficacy and Limitations

Surgical and ablative methods are key in managing visible and histologically confirmed HPV‐related lesions, especially high‐grade CIN 2/3. Common treatments include cryotherapy, laser ablation, and the loop electrosurgical excision procedure (LEEP). Cryotherapy is a low‐cost outpatient procedure that destroys abnormal tissue by freezing but has limited depth, which may lead to incomplete removal and higher recurrence, and it does not provide tissue for histological analysis. Laser ablation offers more precise tissue destruction and better depth control, suitable for complex lesions, but requires specialized equipment and also lacks histological sampling. LEEP, considered the gold standard, removes cervical tissue using an electrically charged loop, allowing for both treatment and histopathological evaluation. However, LEEP carries risks such as bleeding, infection, cervical stenosis, and cervical insufficiency, which can affect fertility and pregnancy outcomes. Additionally, the psychological impact of surgical treatments, especially in young women wishing to preserve fertility, should be addressed [31].

#### 1.4.4. Prophylactic Vaccination

Prophylactic vaccination has emerged as one of the most effective public health strategies for reducing the global burden of HPV‐associated diseases [32]. Currently available vaccines, including Gardasil (quadrivalent and nonavalent) and Cervarix (bivalent), are designed to induce neutralizing antibodies against the L1 capsid protein of high‐risk HPV types—most notably HPV‐16 and HPV‐18, which together account for approximately 70% of cervical cancer cases worldwide. Clinical trials and post‐marketing surveillance data have consistently demonstrated that these vaccines are highly effective in preventing new infections, genital warts, and CIN grades 1–3 (CIN 1–3), particularly when administered before the onset of sexual activity. Long‐term follow‐up studies have shown sustained immunity lasting up to 10–12 years with no evidence of waning protection, highlighting the durability of vaccine‐induced immune responses [33]. Despite these successes, HPV vaccines have key limitations. Primarily, they are prophylactic—not therapeutic, meaning they do not eliminate pre‐existing HPV infections or treat established cervical inflammation, dysplasia, or malignancy. This restricts their utility in the management of women who are already infected, particularly in low‐resource settings where screening and early intervention may be delayed [34]. In summary, while prophylactic HPV vaccination represents a major advance in cancer prevention, its inability to address existing HPV‐induced inflammation and lesions underscores the need for complementary therapeutic strategies.

#### 1.4.5. Phytotherapy and Alternative Medicine: Potential and Pitfalls

Phytotherapy and alternative medicine have gained popularity in recent years as adjunctive or alternative approaches to managing HPV infections, particularly in populations with limited access to conventional healthcare. A variety of herbal extracts, essential oils, and traditional remedies have been proposed based on their antiviral, antioxidant, immunomodulatory, and anti‐inflammatory properties. However, while these therapies offer theoretical and cultural appeal, their clinical application in HPV‐related diseases remains highly controversial [35]. Among the most commonly studied herbal agents are green tea polyphenols (e.g., epigallocatechin‐3‐gallate [EGCG] [36]), curcumin (from turmeric), indole‐3‐carbinol (from cruciferous vegetables), and extracts from medicinal plants such as Echinacea, Astragalus, and *Thuja occidentalis* [37]. Several in vitro studies have demonstrated that these compounds can inhibit HPV oncogene expression (E6 and E7), enhance apoptosis in infected cells, and modulate cytokine profiles. For instance, EGCG has been shown to downregulate HPV16 E6/E7 mRNA expression and restore p53 activity, suggesting a potential anti‐oncogenic effect. Despite these promising laboratory findings, robust clinical evidence is lacking. Most clinical trials involving phytotherapeutic agents suffer from small sample sizes, lack of randomization, short duration of follow‐up, and absence of standardized formulations or dosing. For example, although polyphenon E (a green tea extract) has been approved in some countries for the treatment of external genital warts, its efficacy remains variable and less reliable compared to conventional treatments [38]. Another major concern is the lack of quality control and standardization. Herbal products may vary significantly in composition depending on the source, preparation method, and environmental conditions during cultivation. This inconsistency not only hampers the reproducibility of clinical results but also increases the risk of adverse reactions, toxicity, or interactions with conventional drugs such as antivirals or immunomodulators. In conclusion, while phytotherapy and alternative medicine offer potential adjunctive benefits in HPV management, their role remains limited by insufficient scientific validation, poor standardization, and inconsistent outcomes. This therapeutic gap underscores the need for novel approaches that modulate the inflammatory microenvironment as part of a comprehensive treatment strategy for HPV‐associated diseases. Despite these advances, current therapeutic modalities primarily aim to eliminate HPV‐related lesions or stimulate a general immune response while largely overlooking the role of chronic inflammation in driving disease persistence and progression [39]. As a result, there is a growing recognition that HPV treatment must go beyond lesion ablation and prophylactic vaccination. Instead, novel therapeutic strategies should aim to reprogram the inflammatory microenvironment, promote tissue regeneration, and restore immune homeostasis. Such approaches could enhance viral clearance, reduce recurrence, and mitigate the risk of malignant transformation.

#### 1.4.6. Anti‐Inflammatory Drugs in the Context of HPV

Several anti‐inflammatory agents have been explored in the context of HPV infection, either as adjunct therapies or experimental treatments aimed at modulating the inflammatory microenvironment. Corticosteroids are widely known for their potent anti‐inflammatory and immunosuppressive effects, and they have been utilized in various inflammatory and autoimmune conditions. Corticosteroids, while potent anti‐inflammatories, are generally contraindicated in HPV infections due to their immunosuppressive effects, which may enhance viral persistence and delay epithelial healing. Similarly, systemic immunosuppressants are avoided as they compromise the body’s ability to eliminate HPV‐infected cells [40]. Overall, while current anti‐inflammatory drugs offer partial symptom relief and may influence the HPV‐associated inflammatory response, none directly target the complex immunopathology of persistent HPV infections. Although conventional anti‐inflammatory drugs such as nonsteroidal anti‐inflammatory drugs (NSAIDs), corticosteroids, and immunomodulators have been explored in the management of HPV‐related symptoms, their clinical application remains limited by systemic side effects, nonspecific targeting, and inconsistent outcomes in viral clearance [41]. On one hand, corticosteroids can reduce local inflammation, alleviate pain, and improve short‐term symptomatic relief in cases where HPV lesions cause discomfort or swelling, particularly in the anogenital region [42]. On the other hand, the immunosuppressive nature of corticosteroids may impair the host’s antiviral response, particularly cytotoxic T cell activity and antigen presentation, which are critical for clearing HPV‐infected cells. This suppression may prolong viral persistence, increase the risk of recurrent lesions, and potentially contribute to disease progression in individuals with high‐risk HPV genotypes. Additionally, prolonged corticosteroid use may alter local tissue architecture, delay wound healing, and disturb epithelial regeneration [43]. Consequently, there is a growing demand for the development of novel medical approaches—particularly cell‐based therapies—that can simultaneously and selectively reduce tissue inflammation and promote the repair of inflammation‐induced damage in HPV‐associated conditions. Such strategies aim not only to suppress the chronic inflammatory response triggered by persistent HPV infection but also to restore epithelial integrity and tissue homeostasis, thereby addressing both the symptoms and the underlying pathophysiology of the disease.

### 1.5. Application of Cell‐Based Therapies in HPV‐Related Conditions

Recent advances in immunology and regenerative medicine have led to increasing interest in cell‐based therapies as potential treatments for HPV‐associated diseases, particularly those involving chronic inflammation and neoplastic transformation. While the majority of conventional therapies focus on lesion ablation or antiviral agents, cell therapies offer a more targeted and biologically integrated approach capable of modulating immune responses, suppressing inflammation, and promoting tissue repair.

#### 1.5.1. Potential Role of T Cell‐Based Immunotherapy in Early HPV Infection and Precancerous Lesions

Adoptive T cell therapy (ACT) has primarily been investigated in advanced HPV‐associated malignancies, particularly metastatic cervical cancer. However, increasing attention is being directed toward its potential application in earlier stages of HPV infection, including persistent high‐risk HPV infection and high‐grade CIN 2/3 [44]. The biological rationale lies in the consistent expression of the viral oncoproteins E6 and E7 in HPV‐infected epithelial cells, even at precancerous stages, making them ideal targets for antigen‐specific CTL responses [45]. Unlike prophylactic vaccines, which mainly induce humoral immunity, ACT aims to eliminate infected cells through CTL‐mediated cytotoxicity, potentially reversing early dysplasia and preventing malignant progression. Recent therapeutic strategies emphasize combination immunotherapies, particularly the sequential use of therapeutic HPV vaccines to prime and expand HPV‐specific T cell responses, followed by ACT to enhance CTL activity at mucosal sites of infection. This approach aims to improve immune recognition, sustain effective effector responses, and promote durable viral clearance along with the regression of high‐grade intraepithelial lesions. In addition, advances in cellular engineering have led to the development of off‐the‐shelf allogeneic or universal T cell products, which address the logistical limitations, manufacturing delays, and high costs associated with autologous ACT [46]. These next‐generation immunotherapies are being explored as early intervention strategies to control persistent HPV infection, reduce chronic inflammation, and support epithelial recovery before neoplastic transformation occurs. Nevertheless, further preclinical research and well‐designed clinical trials are required to optimize treatment protocols, evaluate long‐term safety, and confirm therapeutic efficacy across diverse patient populations.

#### 1.5.2. Dendritic Cell‐Based Vaccines in Early HPV Infection and Precancerous Lesions

DCs are professional antigen‑presenting cells that play a central role in initiating adaptive immune responses. DC‑based vaccines have therefore been developed to enhance the presentation of HPV oncoproteins, particularly E6 and E7, to the immune system, with the goal of activating HPV‑specific CTLs capable of eliminating infected or transformed cells before malignant progression [47]. Clinical studies have explored DC vaccines not only in invasive cervical cancer but also in early‑stage lesions such as CIN1–3 and persistent high‑risk HPV infection, where immunological intervention may prevent disease progression. Early‑phase trials indicate that DC vaccines loaded with HPV E6/E7 peptides or RNA can safely induce strong HPV‑specific T‑cell responses and in some cases are associated with lesion regression, reduced viral load, and increased T‑cell infiltration in cervical tissue [48]. DC‑based immunotherapy may also help counteract HPV immune‑evasion mechanisms, including reduced antigen presentation and local immune suppression, by enhancing antigen presentation and costimulatory signaling [49]. However, further research is required to optimize vaccine design, delivery strategies, and dosing and to confirm clinical efficacy through large randomized controlled trials.

#### 1.5.3. Mesenchymal Stem Cells (MSCs) and MSC‐Derived Exosomes

Despite the availability of various conventional therapies, the clinical response to standard treatments in patients with HPV‐associated diseases remains limited. Interventions, such as surgical excision, topical chemotherapeutics, and immunomodulatory agents—including IFNs and imiquimod—often lead to incomplete or short‐lived clinical improvement, with high recurrence rates reported across diverse patient groups [50]. These limitations are particularly evident in cases involving persistent or high‐risk HPV genotypes, where viral immune evasion mechanisms significantly reduce treatment efficacy. As a result of these challenges, interest has shifted toward novel immunotherapeutic strategies that can address both viral persistence and host immune dysfunction. Among the emerging approaches, MSCs have gained attention due to their unique combination of immunomodulatory, anti‐inflammatory, and tissue‐regenerative properties. Their ability to suppress excessive inflammation while promoting the repair of damaged tissues positions them as a potentially transformative therapy for HPV‐related conditions [51]. Based on recent studies, MSCs have been identified as the most accessible and widely studied cell type for therapeutic applications, primarily due to their immunomodulatory capabilities and tissue‐regenerative properties. In vitro investigations have demonstrated that MSCs exhibit promising potential for repairing damaged tissues and modulating immune responses. As a result, they have been proposed as a viable therapeutic option for patients with HPV‐associated diseases [52]. MSCs are a multipotent, nonhematopoietic, and heterogeneous cell population that can be isolated from various tissues, including bone marrow, placenta, umbilical cord, and dental pulp [53]. Their distinctive immunomodulatory and tissue‐regenerative capabilities have garnered considerable interest as therapeutic candidates for a broad spectrum of inflammation‐related disorders. These include acute conditions such as COVID‐19, where MSCs have demonstrated potential in mitigating the cytokine storm and promoting lung tissue repair, thereby improving respiratory function [54]. In cases of myocardial injury, MSCs contribute to cardiac tissue regeneration by modulating inflammation, reducing fibrosis, and enhancing angiogenesis [55]. Similarly, in neural tissue damage, MSCs support neuroprotection and neural repair through the secretion of trophic factors and modulation of local immune responses [56]. Beyond these acute conditions, MSCs are also investigated for their therapeutic potential in chronic autoimmune and inflammatory diseases such as multiple sclerosis, where they may help restore immune tolerance and promote remyelination; Crohn’s disease, through suppression of gut inflammation and promotion of mucosal healing; and systemic lupus erythematosus, by modulating aberrant immune responses and reducing tissue damage [57]. Collectively, these multifaceted properties underscore the versatility of MSCs as promising candidates for cell‐based therapies across a diverse range of inflammatory and degenerative diseases.

Numerous studies have demonstrated the successful administration of MSCs in both pediatric and adult patient populations, with a consistently favorable safety profile [57]. Importantly, these investigations report no significant acute or long‐term toxicities nor any life‐threatening adverse effects associated with MSC therapy. This safety has been observed across a range of clinical contexts and dosing regimens, underscoring the potential of MSCs as a viable therapeutic option. Furthermore, the absence of severe complications supports the continued exploration and application of MSC‐based treatments in various inflammatory, autoimmune, and degenerative diseases, where conventional therapies often fail to provide satisfactory outcomes [58]. However, some studies have reported conflicting outcomes following MSC infusion, which can largely be attributed to the variability in the pharmacological quality of MSC preparations. This variability stems from the absence of standardized protocols for MSC production, inconsistencies in dosing regimens, and donor‐related heterogeneity. Such factors significantly affect the reproducibility and efficacy of MSC‐based therapies [59]. Additionally, there are ongoing concerns regarding potential risks associated with MSC therapy, including unwanted differentiation into nontarget cell types, the possibility of malignant transformation, and adverse immunological reactions. These safety considerations underscore the critical need for rigorous quality control, standardized manufacturing processes, and comprehensive preclinical evaluation to optimize therapeutic outcomes and minimize risks [60]. Addressing these challenges is essential to fully harness the clinical potential of MSCs in treating a variety of diseases. Initially, it was believed that MSCs exert their therapeutic effects primarily through engraftment and direct integration into the target tissue. However, more recent evidence indicates that only a small fraction of administered MSCs actually migrate to and persist within the damaged tissue. Instead, it is now widely recognized that the immunomodulatory and regenerative properties of MSCs are largely mediated by their secreted extracellular factors—particularly extracellular vesicles (EVs), including exosomes [61]. These vesicles carry bioactive molecules, such as proteins, lipids, and nucleic acids, that modulate the local microenvironment and influence immune responses. As a result, the use of MSC‐derived secretome or purified exosomes is increasingly being considered as a promising cell‐free therapeutic alternative, potentially offering similar benefits while minimizing the risks associated with live cell transplantation [61]. Exosomes derived from MSCs (MSC‐exosomes) have garnered increasing attention as a promising cell‐free alternative to their parental MSC counterparts, offering numerous advantages in terms of safety, stability, and therapeutic efficacy. Unlike living cells, MSC‐exosomes are nonproliferative and metabolically inactive, thus eliminating the risk of uncontrolled cell growth or malignant transformation [62]. Moreover, their storage does not require cytotoxic cryoprotectants such as dimethyl sulfoxide (DMSO), which significantly simplifies preservation and transportation procedures while enhancing clinical safety [63]. Due to their metabolic inertness, exosomes are less influenced by the variability of host microenvironments, enabling more consistent functional performance and allowing for better standardization and reproducibility in therapeutic applications [64]. Additionally, the immunological profile of exosomes is favorable; they typically lack MHC molecules, including HLA class I and II antigens, thereby minimizing the risk of eliciting immune responses upon administration to allogeneic recipients [65]. Mechanistically, MSC‐exosomes exhibit the capacity to interact with target cells through receptor‐mediated binding or membrane fusion, facilitating the efficient delivery of their bioactive cargo—including proteins, lipids, mRNAs, and microRNAs—into recipient cells. This unique mode of action supports tissue repair, immune modulation, and regenerative processes without the complications associated with live‐cell transplantation. As such, the MSC secretome, particularly exosomes, represents a robust and clinically viable alternative for the treatment of a broad range of inflammatory, autoimmune, and degenerative diseases [66]. MSC‐exosomes have emerged as critical mediators of these regenerative processes due to their capacity to deliver functional proteins, microRNAs, and other signaling molecules directly into recipient cells. This mode of action may be particularly relevant in the context of HPV‐induced epithelial damage and immune dysregulation. Exosomes also contribute to epithelial repair and barrier restoration by stimulating the proliferation, migration, and differentiation of basal keratinocytes and epithelial progenitor cells. In HPV‐infected tissues, where viral persistence and epithelial dysregulation often coexist, MSC‐exosomes have the potential to reverse the inflammatory microenvironment, reduce fibrosis and tissue scarring, and restore epithelial architecture. These effects are particularly valuable in preventing the progression of low‐grade intraepithelial lesions toward high‐grade neoplasia or invasive cancer [67]. Furthermore, MSC‐exosomes have demonstrated the ability to modulate immune checkpoints and enhance antigen‐presenting cell function, which may indirectly support the clearance of HPV‐infected cells by restoring host immune surveillance. Although these findings remain largely at the preclinical stage, they underscore the promise of MSC‐exosome therapy as a dual‐action platform—targeting both the inflammatory milieu and epithelial damage induced by chronic HPV infection [68]. By inhibiting pro‐inflammatory cytokines such as TNF‐α, IL‐6, and IL‐1β, and enhancing the expression of regulatory mediators like IL‐10 and TGF‐β, MSC‐exosomes help restore immune homeostasis and promote tissue repair [50, 69, 70]. These effects are particularly valuable in chronic HPV infections, where persistent inflammation contributes to cervical epithelial damage and precancerous transformation. Moreover, MSC‐exosomes offer a cell‐free therapeutic platform, reducing the risks associated with live cell therapies while retaining the regenerative and anti‐inflammatory capacity of their parent cells [71]. Importantly, these exosomes can be engineered or enriched with specific microRNAs or proteins that suppress HPV‐related oncogenic pathways or support local immune restoration. This unique convergence between anti‐inflammatory drug goals and MSC‐exosome functionality presents a promising direction for novel HPV therapies—capable of both modulating host inflammation and restoring epithelial homeostasis, without the drawbacks seen in traditional pharmacological agents. Although research directly addressing their role in HPV‐induced inflammation is still limited, emerging data suggest that MSC‐exosomes could provide a novel therapeutic avenue for managing HPV‐associated pathologies, particularly in the female reproductive system.

### 1.6. The Double‐Edged Nature of MSC‐Exosomes in HPV‐Associated Disease

**Figure 2:**
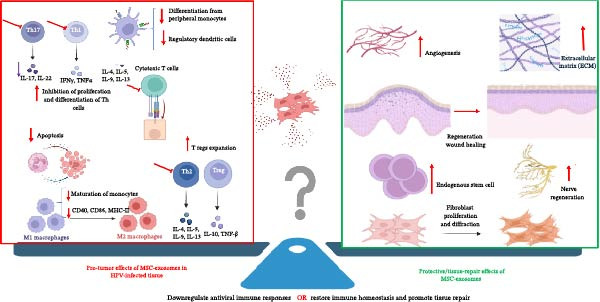
Schematic illustration showing the dual (protective and tumor‐promoting) roles of MSC‐derived exosomes in HPV‐associated disease.

Despite their therapeutic promise, MSC‐exosomes may act as a double‐edged sword in the treatment of HPV‐associated diseases. While they suppress harmful inflammation, they may also downregulate antiviral immune responses, especially CTL activity and antigen presentation pathways. This suppression can impair the clearance of HPV‐infected cells, allowing the virus to persist in epithelial tissues [72]. A central mechanism by which MSC‐exosomes attenuate host immune surveillance against HPV is the suppression of antigen presentation, as they interfere with both the expression and functional capacity of MHC class I and class II molecules on professional APCs, particularly dendritic cells and macrophages [73]. MHC class I molecules are essential for presenting endogenously derived HPV antigens to CD8^+^ cytotoxic CTLs. MSC‐exosomes impair this pathway by suppressing IRF1 through interference with IFN‐γ–STAT1 and NF‐κB signaling, resulting in reduced MHC class I expression and compromised CTL priming. This diminishes the immunological visibility of HPV‐infected cells and weakens cytotoxic immune responses [74]. In parallel, MSC‐exosomes disrupt MHC class II–dependent antigen presentation required for CD4^+^ T cell activation. By downregulating costimulatory molecules such as CD80 and CD86 and suppressing CIITA, the master regulator of MHC class II transcription, MSC‐exosomes impair CD4^+^ T cell priming and supportive antiviral immunity [75]. Moreover, prolonged exposure to MSC‐exosomes in HPV‐infected tissues may inadvertently promote tumor progression by modulating the local microenvironment. MSC‐exosomes can enhance EMT, angiogenesis, and extracellular matrix remodeling, thereby facilitating cellular plasticity, tissue invasion, and neovascular support. These effects are particularly concerning in the context of high‐risk HPV genotypes, such as HPV‐16 and HPV‐18, where viral oncogene activity may synergize with MSC‐exosome–mediated microenvironmental changes to accelerate progression toward cervical neoplasia and cervical cancer [76]. While this phenotype is beneficial for limiting excessive inflammation and tissue damage, it inherently compromises the magnitude and durability of antiviral T cell responses, thereby favoring immune regulation over effective viral clearance. One important mechanism underlying the immunomodulatory effects of MSC‐exosomes is the transfer of immunosuppressive miRNAs to APC, including dendritic cells and macrophages. MSC‐exosomes are enriched in regulatory miRNAs, such as miR‐21, miR‐146a, and miR‐155, which are efficiently internalized by recipient APCs and subsequently modulate key immune signaling pathways and transcriptional programs, including those regulating CIITA and IRF1 [75]. miR‐21 and miR‐146a exert broad immunosuppressive effects by modulating inflammatory signaling cascades and reinforcing a tolerogenic APC phenotype: miR‐21 targets genes such as PTEN and components of the Toll‐like receptor (TLR) signaling cascade, reducing sensitivity to viral molecular patterns [77], while miR‐146a negatively regulates NF‐κB activation by targeting adaptor molecules such as IRAK1 and TRAF6, thereby decreasing the production of pro‐inflammatory cytokines including IL‐6 and TNF‐α [78]. As a result, antigen presentation by MSC‐exosome–conditioned APCs occurs in the absence of adequate costimulatory signaling and within an anti‐inflammatory cytokine microenvironment. This environment promotes suboptimal T cell priming, driving T cell anergy rather than effector differentiation. Concurrent enrichment of IL‐10 and TGF‐β arises primarily from MSC‐exosome–conditioned APCs and M2‐polarized macrophages, which adopt a tolerogenic cytokine profile following exosome uptake [79]. This cytokine milieu skews CD4^+^ T cell differentiation toward a regulatory phenotype, with TGF‐β–dependent FoxP3 induction stabilizing regulatory T cell development and reinforcing Treg‐mediated immune tolerance [64]. Furthermore, the establishment of an immunosuppressive microenvironment—characterized by regulatory T cell expansion, dominance of M2‐polarized macrophages, and sustained downregulation of antigen presentation pathways—facilitates viral immune evasion and promotes the persistence of HPV‐infected cells with oncogenic potential. Over time, this tolerant immune landscape may contribute to the transition from chronic HPV infection to CIN and ultimately to the development of cervical cancer [80]. In contrast to the previously discussed immunosuppressive and potentially tumor‐promoting effects of MSC‐derived exosomes, emerging evidence also supports their therapeutic potential in HPV‐associated malignancies. While the preceding sections focused on mechanisms through which MSC‐exosomes may facilitate immune evasion or support tumor progression, it is equally important to consider their capacity to modulate the tumor‐associated immune and signaling microenvironment in a beneficial manner. In this context, therapeutic strategies capable of reprogramming the tumor‐associated microenvironment have attracted considerable attention, particularly those targeting host regulatory pathways rather than viral components directly. Several biological mechanisms may explain how MSC‐exosomes can indirectly influence the activity of HPV oncoproteins—particularly E6 and E7—thereby affecting HPV‐associated carcinogenesis and potentially promoting tissue restoration. Current evidence suggests that these modulatory effects occur primarily through the regulation of host cellular signaling pathways rather than through the direct inhibition of viral proteins. Most available evidence indicates that these modulatory effects occur primarily through the regulation of host cellular signaling rather than by direct inhibition of viral proteins. First, MSC‐exosomes can suppress pro‑inflammatory signaling by delivering microRNAs such as miR‑146a, which target components of the TLR4/MyD88/NF‑κB pathway. Downregulation of this pathway decreases the production of pro‑inflammatory cytokines (IL‑6, TNF‑α, and IL‑1β), thereby diminishing the chronic inflammatory microenvironment that would otherwise stimulate epithelial hyperproliferation and sustain oncogenic pathways such as STAT3 [81]. Because persistent inflammation favors HPV persistence and enhances the transcriptional activity of viral oncogenes, the downregulation of these signals may help restrict tumor‑promoting conditions. Second, several microRNAs predominantly expressed in MSC‐exosomes such as miR‑34a, miR‑16, and miR‑145 can reinforce tumor‑suppressive pathways governed by p53 and pRb, concurrently downregulating proliferation‑related genes [82]. These effects may partially mitigate the oncogenic functions of HPV E6 and E7, which degrade p53 and inactivate pRb, respectively, thereby reducing the likelihood of uncontrolled epithelial proliferation. In addition, MSC‐exosomes can mitigate oxidative stress by decreasing the generation of ROS and activating Nrf2‑dependent antioxidant defenses. Since HPV‑associated lesions often display elevated oxidative damage and genomic instability, activation of the Nrf2/ARE pathway and subsequent induction of antioxidant enzymes such as HO‑1, SOD, and catalase may protect cells from DNA damage and mutagenic stress [83]. Beyond these indirect anti‑oncogenic effects, MSC‐exosomes exert potent regenerative actions that facilitate tissue repair in HPV‑infected cervical epithelium. In lesions characterized by chronic inflammation and impaired epithelial turnover, MSC‐exosomes deliver a repertoire of regulatory and trophic factors—including EGF, HGF, KGF/FGF7, and TGF‑β—that activate canonical regenerative pathways such as PI3K/AKT, MAPK/ERK, and Wnt/β‑catenin in basal and progenitor epithelial cells [84]. Activation of these signaling cascades promotes cell survival, proliferation, and migration, accelerating the re‑epithelialization and restoration of tissue integrity. MSC‐exosomes further enhance angiogenesis and extracellular matrix remodeling through cargo molecules such as VEGF, angiopoietin‑1, and miR‑126, which stimulate endothelial PI3K/AKT and ERK signaling to promote new vessel formation [85]. Improved vascularization increases oxygen and nutrient delivery to the lesion, supporting sustained tissue regeneration. Moreover, neurotrophic factors and miRNAs such as miR‑133b may attenuate neuroinflammation and support neural network repair within the affected cervicovaginal microenvironment [86]. Modulation of the balance between immunosuppression and immune activation will be key to developing MSC‐based or exosome‐based therapies that effectively treat HPV‐induced lesions while minimizing the risk of viral persistence or malignant transformation. This dual behavior of MSC‐exosomes highlights their complex role in HPV‐associated pathogenesis (Figure 2).

## 2. Clinical Considerations and Future Perspectives

The therapeutic application of MSC‐exosomes presents both great promise and significant caution in the context of viral infections such as HPV. Their potent anti‐inflammatory and immunomodulatory properties make them attractive candidates for controlling excessive immune responses, tissue damage, or chronic inflammation associated with persistent HPV infection. However, this same immunosuppressive potential raises concerns about viral immune evasion, long‐term persistence, and even oncogenic progression, particularly in patients already infected with high‐risk HPV strains.

Given this double‐edged nature, future clinical strategies involving MSC‐exosomes must be approached with precision and individualized risk assessment. Several key clinical variables must be considered [87].

Dose optimization is critical, as excessive amounts of exosomes could amplify immunosuppressive effects and unintentionally support viral latency or tumor microenvironment formation. Conversely, insufficient dosing may fail to exert therapeutic effects.

The timing of administration must align with the immunological state of the patient. For instance, applying MSC‐exosomes during early HPV infection or during active viral replication might promote persistence, whereas their use post‐clearance (to manage residual inflammation or epithelial damage) may offer safer benefits.

The route of delivery significantly impacts biodistribution, cell targeting, and immune interactions. Localized delivery (e.g., intravaginal or intra‐lesional) may reduce systemic immune suppression and limit off‐target effects compared to systemic infusion.

To overcome the risks associated with unmodified MSC‐exosomes, several next‐generation strategies are being investigated:

– Engineered exosomes with selectively loaded cargo (e.g., anti‐inflammatory cytokines but lacking immunosuppressive miRNAs like miR‐21 or miR‐146a) offer greater safety and disease specificity.
– Combination therapies, where MSC‐exosomes are co‐administered with antiviral agents (e.g., IFN‐α, immune checkpoint inhibitors, or HPV vaccines), may help maintain antiviral surveillance while benefiting from the regenerative effects of exosomes.
– Targeted exosome delivery systems, using surface modifications or ligand‐conjugation, can enhance tissue‐specific uptake and reduce undesired immune modulation elsewhere.
– Importantly, well‐designed in vivo models and phase I/II clinical trials are urgently needed to assess the safety profile, pharmacodynamics, and therapeutic window of MSC‐exosomes in patients with active or latent HPV infection. Additionally, biomarker development will be crucial to identify patient subgroups most likely to benefit from such therapies—such as those with resolved infection but ongoing cervical inflammation or those at high risk of immune‐mediated epithelial damage.

## 3. Conclusion

In conclusion, while MSC‐exosomes hold significant therapeutic potential, their use in HPV‐related disease requires caution. A precision medicine approach will be essential to maximize benefits while limiting the risks of viral persistence and malignant progression. Given that the biological effects of exosomes are largely determined by their internal cargo, including miRNAs, regulatory RNAs, and immunomodulatory proteins, engineered MSC‐exosomes carrying selective antiviral or immunostimulatory molecules—such as miRNAs targeting HPV E6/E7 transcripts or modulators of key inflammatory pathways—may represent a promising strategy to reprogram the local microenvironment. Such an approach could shift the balance from HPV‐induced immunosuppression toward effective antiviral immunity and directly address the double‐edged nature of immune modulation.

**Nomenclature**
ACT: Adoptive T cell therapy
APC: Antigen‐presenting cell
CIN: Cervical intraepithelial neoplasia
CIITA: Class II transactivator
COX‐2: Cyclooxygenase‐2
CIN 1–3: Cervical intraepithelial neoplasia grades 1–3
CTL: Cytotoxic T lymphocyte
DC: Dendritic cell
DNA: Deoxyribonucleic acid
dsRNA: Double‐stranded RNA
DMSO: Dimethyl sulfoxide
EMT: Epithelial–mesenchymal transition
EGCG: Epigallocatechin gallate
EV: Extracellular vesicle
HPV: Human papillomavirus
IEN: Intraepithelial neoplasia
IFN: Interferon
IFN‐α: Interferon‐alpha
IFN‐β: Interferon‐beta
IL‐1β: Interleukin‐1 beta
IL‐6: Interleukin‐6
IL‐10: Interleukin‐10
IRF: Interferon regulatory factor
IRF1: Interferon regulatory factor 1
ISG: Interferon‐stimulated gene
JAK‐STAT: Janus kinase–signal transducer and activator of transcription
L1: Major capsid protein L1
LMP: Low molecular weight protein
LEEP: Loop electrosurgical excision procedure
MHC‐I: Major histocompatibility complex class I
miRNA: MicroRNA
MSC: Mesenchymal stem cell
MSC‐exosomes: Mesenchymal stem cell‐derived exosomes
MS: Multiple sclerosis
NF‐κB: Nuclear factor kappa‐light‐chain‐enhancer of activated B cells
NO: Nitric oxide
NK: Natural killer
NSAID: Nonsteroidal anti‐inflammatory drug
pRb: Retinoblastoma protein
PRR: Pattern recognition receptor
PTEN: Phosphatase and tensin homolog
ROS: Reactive oxygen species
SLE: Systemic lupus erythematosus
STAT: Signal transducer and activator of transcription
STI: Sexually transmitted infection
TAP: Transporter associated with antigen processing
TGF‐β: Transforming growth factor‐beta
Th: T Helper cell
TNF‐α: Tumor necrosis factor‐alpha
TLR7: Toll‐like receptor 7
TLR8: Toll‐like receptor 8
Treg: Regulatory T cell
TRAF6: TNF receptor‐associated factor 6
VEGF: Vascular endothelial growth factor.

## Author Contributions

All authors contributed substantially to the conception, design, writing, and critical revision of the manuscript.

## Funding

This research did not receive any specific grant from funding agencies in the public, commercial, or not‐for‐profit sectors.

## Disclosure

All authors have read and agreed to the published version of the manuscript, provided final approval of the manuscript for publication, and accept responsibility for the integrity and accuracy of its content. No unacknowledged individuals or third‐party services contributed to the research design, data analysis, interpretation, or manuscript preparation. All contributions have been appropriately disclosed and acknowledged within the manuscript. All scientific content was reviewed and verified by the authors.

## Ethics Statement

This study is a narrative review without involving humans or experimental animals.

## Conflicts of Interest

The authors declare no conflicts of interest.

## Data Availability

The data that support the findings of this study are available from the corresponding author upon reasonable request.
